# Real-World Clinical Outcomes of Neoadjuvant Platinum-Based Chemotherapy with Nivolumab in Non-Small Cell Lung Cancer

**DOI:** 10.3390/jcm13216568

**Published:** 2024-10-31

**Authors:** Walid Shalata, Sameh Daher, Natali Maimon Rabinovitch, Sivan Shamai, Waleed Kian, Ilit Turgeman, Yulia Dudnik, Olga Kazareen, Yulia Rovitsky, Edmond Sabo, Dan Levy Faber, Ronen Galili, Ory Wiesel, Konstantin Baranovsky, Abed Agbarya

**Affiliations:** 1 The Legacy Heritage Cancer Center, Dr. Larry Norton Institute, Soroka Medical Center, Ben-Gurion University, Beer Sheva 84105, Israel; 2 Medical School for International Health, Faculty of Health Sciences, Ben Gurion University of the Negev, Beer Sheva 84105, Israel; 3 Thoracic Cancer Unit, Cancer Division, Rambam Health Care Campus, Haifa 31096, Israel; 4 Lung Oncology Service, Division of Oncology, Meir Medical Center, Kfar Saba 44281, Israel; 5 Ichilov Medical Center, Tel-Aviv 64239, Israel; 6 Assuta Medical Center, Ashdod 77600, Israel; 7 Linn Medical Center, Haifa 35254, Israel; 8 Hillel Yaffee Medical Center, Hadera 38203, Israel; 9 Department of Pathology, Carmel Medical Center, Haifa 34362, Israel; 10 Department of Thoracic Surgery, Carmel Medical Center, Haifa 34362, Israel; 11 Rappaport Faculty of Medicine, Technion-Israel Institute of Technology, Haifa 35254, Israel; 12 Division of Thoracic and Esophageal Surgery the Cardiovascular Center, Tzafon Medical Center, Poriya 15208, Israel; 13 Department of Oncology, Bnai-Zion Medical Center, Haifa 31048, Israel

**Keywords:** neoadjuvant, chemotherapy, immunotherapy, non-small cell lung cancer, resection surgery, real-world clinical outcomes

## Abstract

**Background:** Lung cancer is among the most prevalent and serious forms of cancer, characterized by an allogenic phenotype that presents significant therapeutic challenges. **Materials and Methods:** We analyzed medical records from January 2022 to August 2023, focusing on individuals aged 18 and older diagnosed with resectable NSCLC who received neoadjuvant chemo-immunotherapy prior to surgical intervention. **Results:** The cohort comprised 56 patients, predominantly smokers (95%) and male (74%), with 80% presenting the disease at stage III. Of the participants, 44 underwent surgery, with 95% receiving lobar resection. Clinical assessments via PET-CT imaging revealed an 86% rate of response or disease stabilization, while pathological evaluations showed complete and major pathological responses in 61% of cases. **Conclusions:** This real-world data supports the safety and efficacy of incorporating immune checkpoint inhibitors in the neoadjuvant treatment of NSCLC, followed by surgical resection.

## 1. Introduction

Lung cancer is one of the most commonly diagnosed malignancies worldwide [1,2]. In the United States, 238,340 new cases of lung cancer were documented, and 127,070 deaths were reported, showing the high morbidity and mortality of this disease [3]. More than two-thirds of the cases are diagnosed in individuals over the age of 65–70, while roughly up to 5% are diagnosed in individuals under 40–45 years old. The heightened morbidity and mortality rates associated with NSCLC arise from its frequent diagnosis at progressive stages. Nearly 65–70% of patients are diagnosed in advanced stages (III/IV) [4,5,6,7]. This late detection at an advanced stage of an aggressive tumor, with a variety of phenotypes and acquired resistance to treatment, contributes to the poor prognosis of lung cancer in the majority of cases [3,4].

Lung cancer is histologically divided into small cell lung cancer, which comprises approximately 15–20% of cases, and non-small cell lung cancer (NSCLC), which accounts for almost 80–85% of cases [4,5,6,7]. Diagnosis of NSCLC is further subtyped into three main categories: adenocarcinoma (45–50% of cases), large-cell carcinoma (10–15% of cases), squamous cell carcinoma (30–35% of cases), and others (almost 5% of cases) [4]. The TNM system classifies and categorizes different stages of the disease [4]. NSCLC treatment is contingent upon the cancer stage and the patient’s health. Understanding staging is crucial for determining whether tumor removal is viable. Tumors at stages I and II, which are diagnosed in almost 32% of non-metastatic resectable lung cancer patients, signify a localized disease. These early stages are usually eligible for resection without metastatic concerns, while stages IIIC and IV preclude resection. Stage IIIA presents a unique situation: T3N0M0 tumors are resectable, but T3N2M0 tumors are not [3,5,6]. The 5-year survival rate for clinical stage IA1 and IIB were reported to be 92% and 53%, respectively [5,6]. However, for locally advanced stage III lung cancer, which accounts for 25% of the patients, the 5-year survival rate range is 13–36% [5,6]. Among 70% of resectable stage IB-IIIA patients, recurrence was observed both loco-regionally and distantly (33% and 67%, respectively) at a median follow-up of 4.5 years [8,9].

Systemic treatment strategies for resectable NSCLC follow three main approaches. The preoperative approach involves the administration of neoadjuvant systemic therapy prior to surgery. The postoperative approach involves applying surgery first and then systemic therapy. The third approach of perioperative therapy combines both pre and postoperative modalities, i.e., systemic therapy, followed by surgery and, later, another round of systemic therapy [2,10]. Immunotherapy consensus for early-stage NSCLC patients has recently been published [11]. Neoadjuvant chemotherapy administered to NSCLC patients was shown to have improved progression-free survival (PFS) by the addition of an immunotherapeutic agent, nivolumab, which is an immune checkpoint inhibitor. Phase I/II and phase III clinical trials NADIM II and Checkmate 816, respectively, demonstrated the effectiveness of this combination therapy [2,10,12]. The impressive results of the phase III Checkmate 816 trial have made a revolution in the treatment of resectable disease NSCLC, and neoadjuvant chemotherapy/immunotherapy (CT/IO) has become a crucial part of the multi-disciplinary treatment of this disease [13].

The goal of adjuvant therapy is to minimize the risk of relapse by eradicating minimal residual disease [14]. Patients with completely resected stage IB-IIIA NSCLC disease had documented recurrence in 33% of cases (out of 831 patients in France, Germany, and the UK). A total of 68% of them had distant metastasis involvement [15,16]. The risk of distant metastases was higher than local and regional risk, indicating the need for earlier improved systemic control, such as preoperative neoadjuvant effective therapy [17]. Lung cancer disease was shown to have a low response rate to standard of care chemotherapy and radiation in addition to challenging treatment for metastases [4,18]. Adjuvant chemotherapy has increased the overall survival rate by 5% [13]. However, an NSCLC Meta-analysis Collaborative Group publication on neoadjuvant chemotherapy found that it led to a 5% increase in 5-year survival benefit [19]. These clinical outcomes have called for the development of better adjuvant treatments.

Clinical trials KEYNOTE 91 and IMpower 010 reported novel treatment approaches by the administration of immunotherapy [20,21,22]. Standard of care treatment for patients diagnosed with metastatic NSCLC incorporates immune checkpoint inhibitors (ICIs) [7]. Nivolumab, an immune checkpoint inhibitor, blocks PD-L1, a protein that is expressed by tumor cells, thus enabling T cells to kill the cancer cells [23]. Nivolumab was approved by the Food and Drug Administration in 2022 for adult NSCLC patients as part of a neoadjuvant setting in combination with platinum-doublet chemotherapy [7]. ICIs administered both at neoadjuvant and adjuvant venues display their efficient mode of action by showing better clinical outcomes than without immunotherapy [7]. Neoadjuvant immunotherapy triggers a T cell anti-tumor response, aiming to reduce its size before surgery [7,10]. This may lead to resection surgery of a smaller magnitude, i.e., lobectomy vs. pneumonectomy. In addition, the pathologic response evaluates the neoadjuvant treatment on tissue removed during the operation [14]. The objective of adjuvant immunotherapy is to eliminate undetectable “micro metastatic” residual tumor cells that may exist in lymph nodes, blood vessels, or lymphatic vessels after surgery [10].

In the perioperative approach, the role of adjuvant therapy is to prevent recurrence by inducing an anti-metastatic environment [7]. The KEYNOTE-671 clinical trial results describe pembrolizumab administration in a perioperative setting, both as a neoadjuvant agent combined with chemotherapy and as an adjuvant agent for early NSCLC [24]. This potent immune checkpoint blocker had improved event-free survival and pathological complete response (pCR) in comparison to the neoadjuvant therapy modality.

The aim of the study was to check the efficacy of the combination of neoadjuvant therapy on the major pathological response (MPR), which is defined as less than 10% residual viable tumor after neoadjuvant therapy, and pCR, defined as no viable residual tumor.

## 2. Materials and Methods

### 2.1. Study Design

This is a real-world retrospective multicenter observational study. Fifty-six NSCLC patients’ data, retrieved from medical records files of nine medical centers in Israel (Soroka Medical Center, Rambam Health Care Campus, Meir Medical Center, Ichilov Medical Center, Assuta Medical Center, Linn Medical Center, Hillel Yaffee Medical Center, Carmel Medical Center, and Bnai-Zion Medical Center) composed the study cohort.

### 2.2. Patients Enrolled

Inclusion criteria were 18 years of age or older, the patients being diagnosed with lung cancer NSCLC in early stages (IB-IIIA), and Eastern Cooperative Oncology Group (ECOG) performance-status scores of 0 to 4 (on a 4-point scale, with higher scores indicating increasing disability). Each of the studied patients was presented to and discussed with a multidisciplinary medical team when admitted to the medical centers’ oncology institute as per the standard protocol. This team included a medical oncologist, a thoracic surgeon, a pulmonologist, a pathologist, an imaging physician, a nuclear medicine physician, and a radiation oncologist. This multidisciplinary team evaluated the patient’s status, pathology findings, and imaging reports, after which each patient was assigned a primary physician, an oncology specialist responsible for the treatment plan. The patients received a neoadjuvant treatment of combined chemotherapy and immunotherapy with nivolumab, followed by resection surgery. The patients included in the study had not received any previous systemic therapy for any other oncologic disease.

The exclusion criteria comprised other treatment regimens and the presence of epidermal growth factor receptor (EGFR) and anaplastic lymphoma kinase (ALK) mutations within the tumor.

Each institutional review board (IRB) approved the study. The IRB waived patients’ consent due to the retrospective nature of the study.

### 2.3. Treatment Administered

The planned systemic treatment consisted of platinum-based chemotherapy with nivolumab 360 mg in 3-week intervals for up to 3 cycles in total.

#### 2.3.1. Non-Squamous Lung Cancer Patients Received 3 Cycles of Chemotherapy of the Physician’s Choice

Cisplatin (75 mg per square meter of body surface area) or carboplatin (area under the concentration–time curve, 4–5 mg per milliliter per minute, depending on the performance status), plus pemetrexed (500 mg per square meter), plus nivolumab at a fixed dose of 360 mg every three weeks for a total of three cycles. Prior to pemetrexed treatment, premedication of folic acid, vitamin B12, and glucocorticoids was administered to all patients.

#### 2.3.2. Squamous Cell Carcinoma Lung Cancer Patients Received 3 Cycles of Chemotherapy of the Physician’s Choice

Cisplatin (75 mg per square meter of body surface area) or carboplatin (area under the concentration–time curve, 6.5 or 4 mg per milliliter per minute, depending on the performance status), plus paclitaxel (175 mg per square meter) or gemcitabine (1000 mg per square meter, on days 1 and 8) plus nivolumab at a fixed dose of 360 mg every three weeks for a total of three cycles.

### 2.4. Surgery

All patients underwent a positron emission tomography-computed tomography (PET-CT) imaging for disease restaging purposes 3 weeks after the last dose of neoadjuvant treatment. The multidisciplinary team held a discussion prior to the decision for surgery. The Response Evaluation Criteria in Solid Tumors (RECIST) served for disease evaluation by the treating physician [25]. Surgery was performed 4–8 weeks after the last dose of neoadjuvant therapy cycles.

A board-certified thoracic surgeon performed all of the surgeries in a minimally invasive fashion. Standard surgery included lobar resection with mediastinal lymph node dissection. Patients’ admission either to the intensive care unit or to the thoracic surgery unit following the surgery depended on the complexity of the procedure and the hospital protocols. Two weeks after discharge, the thoracic surgery clinic followed up the patients. Follow-ups continued both by the thoracic surgeon and by the oncologist.

### 2.5. Molecular Profiling

Next-generation sequencing (NGS) genetic testing was performed on biopsy samples for the following oncogenes: epidermal growth factor receptor (EGFR), anaplastic lymphoma kinase (ALK), and ROS proto-oncogene 1 (ROS-1). Also, programmed death ligand 1 (PD-L1), microsatellite instability, and tumor burden were evaluated.

Immunohistochemistry assessed PD-L1 protein expression. Tumor proportion score (TPS) calculated the percentage of viable tumors showing membrane staining [26].

### 2.6. Data Analysis

Descriptive statistics were used to calculate frequency (*n*) and percentage for all the parameters of the study. The median and range present the age, follow-up, and days in hospital variables. Fisher’s exact test was used to assess the association between PD-L1 status and the response to treatment.

## 3. Results

### 3.1. Patient Characteristics

Fifty-six patients were included in the study (Table 1). The median follow-up duration was 10 months (range 3–17 m). The median age of the study cohort participants was 65 years old, and 74% were male. A total of 70% of patients reported current smoking.

A total of 51% of lung cancer tumors had a histological type of adenocarcinoma. Stage III disease had an 80% prevalence in the study cohort.

All samples underwent molecular profiling. Analysis of oncogenes ALK, ROS, and EGFR were negative. PD-L1 analysis was performed in 48 patients, and 70% scored > 1%. Other co-mutations detected were KRAS, STK11, p53, and BRCA.

### 3.2. Treatment

#### Neoadjuvant Therapy

Taxol^®^ (paclitaxel) and Alimta^®^ (pemetrexed) are the most commonly administered platinum-based drugs, given to 48% and 46% of cases, respectively (Table 2).

Twice as many patients reported adverse events of any grade following chemotherapy (*n* = 38) than by immunotherapy (*n* = 17) (Table 3). Most adverse events were described as grades 1, 2. Moreover, three patients needed to discontinue the treatments.

### 3.3. Response to Treatment

**Figure 1:**
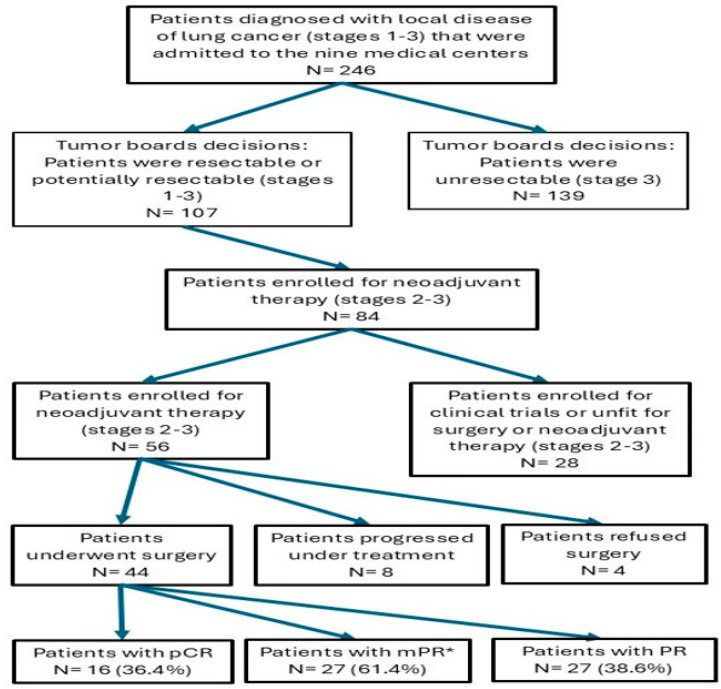
Flow diagram of the study summarizing patient selection and key outcomes, including pathological results. Abbreviations: N, number; Pathological complete response, pCR; Major pathological response, MPR; Partial response, PR. * mPR is calculated including the pCR results.

Post-treatment staging was performed using PET-CT. Response to treatment was detected in forty (72%) patients, while tumor progression was evident in eight (14%) cases (Table 4). All the non-responders were evaluated as disease progression to either stage IIIB (*n* = 5) or non-operable stage IIIA (*n* = 3). These patients expressed a progressive disease as local progression, but without distant metastasis. Four out of forty-eight responders were not operated due to patients’ refusal and loss of follow-up. Among the forty-four operated patients, pathological complete response was found in sixteen (36.4%), major pathological response was found in twenty-seven (61.4%), and partial pathological response in seventeen (38.6%) (mPR is calculated including the pCR results) (Table 4 and Figure 1). There are no statistical differences in the response to treatment rate according to the type of histology, TMB, and gender (*p* = 0.68, *p* = 0.67, and *p* = 0.67, respectively).

### 3.4. Surgery Results

Forty-four of the patients’ cohort medical records files documented surgery data (Table 4) (four patients refused surgery for personal reasons). Minimally invasive thoracoscopic surgery was performed in forty-two (95%) cases. Lobar resection was performed in forty-three patients (98%), and pneumonectomy was performed in one patient (2%). All tumors were resected with an appropriate margin (R0). Post-surgery complications were recorded in five (11%) patients, three of them had prolonged air leak (6%), one had pneumonia (2%), and one had surgical site infection (2%).

The association between PD-L1 status and the treatment response was analyzed using Fisher’s exact test, comparing the rates of the responders (*n* = 48) and non-responders (*n* = 8) in the current study. A total of 17% of responders and 75% of non-responders presented a negative PD-L1 status (*p*-value = 0.0019). Conversely, 69% of responders and 12.5% of non-responders expressed a positive PD-L1 status (*p*-value = 0.004). Of note, 14% of responders and 12.5% of non-responders had unknown PD-L1 status. These findings underscore the significance of PD-L1 status as a predictive biomarker for treatment response, emphasizing its potential utility in guiding therapeutic decisions for patients undergoing immunotherapy. Descriptive statistics in terms of median, percentages, and ranges were calculated for all parameters in the study. Median and ranges were calculated for age, follow-up time, and days in hospital variables. Fisher’s exact test was performed to test the association between smoking status, histology, gender, PD-L1, and TMB according to the response to treatment rate. A *p* < 0.05 was considered significant (Table 5).

## 4. Discussion

Lung cancer is the leading cause of cancer-related deaths globally, claiming an estimated 1.8 million lives in 2020. Traditionally, the standard treatment for resectable NSCLC involved lobectomy followed by systemic adjuvant therapy. However, recent research has highlighted the benefits of neoadjuvant combination therapy (chemo-immunotherapy), ushering in a new perspective. Neoadjuvant treatment presents an alternative avenue to enhance survival rates in patients with resectable NSCLC. This approach offers several potential advantages, including tumor downstaging and early management of micrometastases, facilitating complete resection and improving tolerability [27,28].

The present retrospective multicentric study investigated patients diagnosed with advanced but resectable NSCLC. The present research aimed to assess the efficacy of the combination therapy, i.e., chemo-immunotherapy, in real-world data. Treatment of early-stage NSCLC is complex and involves several modalities due to a variety of factors, such as genetic mutations and other clinical biomarkers [29].

Following major clinical trials and approval by the FDA, the NCCN guidelines [Version 1.2024] for non-small cell lung cancer recommend perioperative systemic therapy for patients who are candidates for immune checkpoint inhibitors. The NCCN guidelines provide neoadjuvant and adjuvant updates on an ongoing basis [30,31]. Nivolumab and platinum-doublet chemotherapy are suggested for neoadjuvant modality. Pembrolizumab and cisplatin-based doublet chemotherapy for 4 cycles, followed by surgery and adjuvant pembrolizumab every 3 weeks for up to 13 cycles [30,31].

Early stages of NSCLC are treated with a pre-surgery approach, resection, and post-operative therapy. The CheckMate 77T and KEYNOTE 671 trials [24] reported that perioperative tri-phasic treatment improved survival benefits. The KEYNOTE 671 clinical trial included a multimodality approach of both neoadjuvant and adjuvant therapies. These treatments used immune checkpoint inhibitor pembrolizumab and provided more benefits than a single modality approach [24]. Immunotherapy reached wide international consensus for NSCLC. The addition of immunotherapy, e.g., nivolumab as an agent in neoadjuvant treatment along with platinum-based chemotherapy, to NSCLC patients presented in the current study is in line with the published results of the clinical trials [12,32]. The real-world outcomes of nine medical centers in Israel demonstrated a 36% pathological complete response comparable to NADIM II trial results from hospitals in Spain, which achieved a 37% pathological complete response. Moreover, the sample size of the study cohort was similar: 56 patients were included in the Israeli study and 57 were found eligible in Spain [12].

We observed higher rates of partial clinical responses (36%) and major pathological responses (25%) compared to those reported in clinical trials. This difference may be attributed to the fact that 82% of our patients were in stages IIB and IIIA. Additionally, over 70% of our patients tested positive for PD-L1, which is known to be a surrogate marker for a better response to immunotherapy. This is higher than the 55–60% positivity rates typically reported in clinical trials. Furthermore, 70% of our patients were current smokers, a group known to generally have better responses to immunotherapy compared to former or never-smokers. A systematic review and meta-analysis support this, showing that overall response rates to immunotherapy were significantly higher in current and former smokers compared to never-smokers (36% vs. 26% vs. 14%; *p* = 0.02), with even greater differences among patients with PD-L1 tumor proportion score (TPS) ≥ 50% (current smokers 58% vs. never-smokers 19%; *p* = 0.03) [32].

A recurrence rate of 23.6% has been reported for patients with pathologic complete response after neoadjuvant therapy for locally advanced NSCLC [17]. Pathologic complete response is observed upon not finding viable tumor cells at the primary tumor site or lymph nodes [33]. Provencio et al. reported treatment of stage III NSCLC with a perioperative approach, which included a neoadjuvant step of nivolumab and platinum-based chemotherapy followed by surgery and an adjuvant therapy of nivolumab for 6 months [12]. This clinical trial (NADIM II) resulted in 37% of patients presenting a pathological complete response and longer survival than chemotherapy. That study showed evidence of the synergistic effect of immunotherapy combined with chemotherapy [12].

Currently, there are four main therapies that may be utilized as neoadjuvant or perioperative treatments: pembrolizumab from the KEYNOTE-671 trial [24], nivolumab from the CheckMate 816 trial [13], toripalimab from the Neotorch trial [33], and durvalumab from the AEGEAN trial. Several notable differences emerge upon comparing the current study results with findings from the other four main trials assessing the efficacy of neoadjuvant chemo-immunotherapy (Table 6). The present study observed a pathologic complete response (pCR) rate of 36%. Contrarily, the CheckMate 816 trial, which administered chemotherapy plus nivolumab for three cycles, reported a pCR rate of 24%. The AEGEAN trial, employing chemotherapy plus durvalumab for four cycles, reported a pCR rate of 17.2%. Similarly, the KEYNOTE-671 trial, utilizing chemotherapy plus pembrolizumab for four cycles, reported a pCR rate of 18.1%. Notably, the Neotorch trial, combining chemotherapy plus toripalimab for three cycles, reported a pCR rate of 24.8%. Regarding the rate of major pathologic response (MPR), the present study reported a high rate of 61%. In comparison, the CheckMate 816 trial reported a MPR rate of 36.9%, the AEGEAN trial reported a rate of 33.3%, the KEYNOTE-671 trial reported a rate of 30.2%, and the Neotorch trial reported a rate of 48.5%.

In terms of the incidence of grade ≥ 3 adverse events related to immunotherapy, the current study reported a rate of 12%. In contrast, the Neotorch trial reported a significantly higher rate of 63.4% adverse events. Nevertheless, 0.5% of fatal adverse events relate to toripalimab administration. The CheckMate 816 trial reported a rate of 33.5% adverse events; however, without treatment-related deaths. The AEGEAN trial reported a rate of 42.4% adverse events, with 5.7% of fatal adverse events related to durvalumab administration. The KEYNOTE-671 trial reported a rate of 12.6% adverse events, with 1% death attributed to adverse events. In the current study, 5.17% of the cases discontinued the treatment due to adverse events. This rate was lower compared to the AEGEAN trial (12%), the Neotorch trial (9.4%), the KEYNOTE-671 trial (12.6%), and the CheckMate 816 trial (10.2%). In addition, in our study, the rate of treatment discontinuation was 5.17%, which is much lower than the one observed in clinical trials (10–12%). This reduced rate may be attributed to the clinical experience of our treatment team and their heightened awareness of the need for close monitoring gained through extensive use of immunotherapy and chemo-immunotherapy. Promptly diagnosing and effectively managing adverse events likely contributed to better patient outcomes and reduced the need for discontinuation.

The limitations of the current study are attributed to the retrospective and multicenter nature of the investigations. Medical records file collection showed that out of 56 patients eligible as per inclusion criteria, 44 had undergone surgery procedures, which reduced the sample size of the study cohort. Another limitation is that the majority of the study population consisted of stage II and III patients, without the inclusion of stage I patients. This could potentially impact the results of the study, considering that patients with more advanced diseases may derive greater benefit from such an approach. Another limitation is that the study is retrospective. Retrospective studies rely on data collected from past records, which may introduce biases or limitations in data collection and analysis. Another limitation of this study is the short observation period, which hinders the accurate calculation of event-free survival (EFS). A limited timeframe may not capture the full spectrum of disease progression, potentially leading to an underestimation of EFS. Consequently, the findings may not fully reflect the long-term efficacy of the treatment being assessed.

Future directions in lung cancer treatment research could comprise molecular assays developed for biomarkers as predictors of specific targeted therapies encompassing all stages of NSCLC.

## 5. Conclusions

The current study demonstrates the advantages of neoadjuvant chemo-immunotherapy for NSCLC patients with resectable disease. These findings align with a recently published clinical trial report, reinforcing the efficacy of neoadjuvant chemo-immunotherapy followed by surgery. Importantly, the approach was found to be safe and feasible, with no significant complications reported.

## Figures and Tables

**Table 1:** Demographic and clinical characteristics of the study population (*n* = 56).

Characteristic	Frequency (*n*)	Percentage (%)
Age, (years), median [range]	65 [51–79]	
Gender		
Female	15	26
Male	41	74
Baseline conditions	43	77
COPD/IHD	34	56
Smoking habits		
Current	39	70
Past	10	18
Light	4	7
Never	3	5
Follow-up (months), median [range]	10 [3–17]	
Histology		
Adenocarcinoma	29	52
Squamous cell carcinoma	22	39
Other	5	9
DiseasesStage		
IIB	11	20
IIIA	35	62
IIIB	10	18
Molecular profile of EGFR, ALK, ROS1-performed	56	100
TMB-performed	42	75
MSI-performed	41	73
PD-L1-performed	48	86
Time till results (days), median [range]	8 [4–22]	
PD-L1 expression level		
>1%	34	70
<1%	14	30
Other mutations checked		
KRAS	3	5
STK11	1	2
P53	3	5
BRCA	1	2

Abbreviations: COPD, chronic obstructive pulmonary disease; IHD, ischemic heart disease; EGFR, epidermal growth factor receptor; ALK, anaplastic lymphoma kinase; ROS, ros-proto oncogene1; PD-L1, programmed death ligand 1; TMB, tumor mutational burden; MSI, microsatellite instability; KRAS, Kirsten rat sarcomas viral oncogene; STK, serine/threonine kinase 11; BRCA, breast cancer gene. Notes: PD-L1 score < 1% is considered negative. PD-L1 score > 1% is considered positive.

**Table 2:** Treatment: platinum-based chemotherapy used in the study population (*n* = 56).

Platinum-Based Combination	Patients *n* (%)	No. of Cycles (No. of Patients, Percentage)
Paclitaxel	27 (48)	1 (2 patients, 4%)
Pemetrexed	26 (46)	2 (1 patient, 2%)
Gemcitabine	2 (4)	3 (40 patients, 70%)
Etoposide	1 (2)	4 (13 patients, 23%)

**Table 3:** Treatment reported toxicity and adverse events *n* (%).

Treatment	Any Grade	Grades 1, 2	Grades 3, 4
Chemotherapy, *n* (%)	38 (68%)	31 (81%)	7 (18%)
Immunotherapy, *n* (%)	17 (30%)	15 (88%)	2 (12%)
Dose change			
Dose interruption	3 (5.17%)		
Dose reduction	1 (1.72%)		
Discontinuation	3 (5.17%)		
Adverse events ^a^			
Diarrhea	13 (23%)	11(84%)	2 (15%)
Nausea/vomiting	14 (25%)	14 (100%)	
Weakness	29 (52%)	25 (86%)	4 (13%)
Neuropathy	11 (20%)	9 (82%)	2 (18%)
Thyroid	14 (25%)	13 (93%)	1 (7%)
Skin	12 (21%)	12 (100%)	

Notes: ^a^ any grade adverse events rates were calculated as percentage of total cohort *n =* 56. Grades 1, 2 and grades 3, 4 toxicity rates were calculated as percent of the “any grade” category.

**Table 4:** Clinical description of surgery and response to treatment.

Procedure	Patient Frequency *n*, (Percentage %)
Surgery	44 (78%)
Thoracoscopy	42 (42/44, 95%)
Open surgery	2 (2/44, 5%)
Resection	
Lobectomy	43 (98%)
Pneumonectomy	1 (2%)
Days in hospital, median [range]	4 [2–29]
Complications	5 (5/44, 11%)
Prolong air leak	3 (3/44, 6%)
Pneumonia	1 (1/44, 2%)
Surgical site infection	1 (1/44, 2%)
Clinical response by PET-CT	
Complete response	6 (11%)
Partial response	34 (61%)
Stable disease	8 (14%)
Tumor progression	8 (14%)
Surgery	44 (79%)
Pathological response	
Complete response	16 (36.4%)
Major pathologic response	27 (61.4%)

Abbreviations: PET-CT, positron emission tomography-computed tomography; *n*, number.

**Table 5:** The clinical biomarker for response by Fisher’s exact test.

	No Response; *n =* 8	Response; *n* = 48	*p*-Value
PD-L1 status			
Negative ^a^	6/8 (75%)	8/48 (17%)	0.0019
Positive ^b^	1/8 (12.5%)	33/48 (69%)	0.004
Unknown	1/8 (12.5%)	7/48 (14%)	1.00
Smoking status			
Current and past	3/8 (37.5%)	46/48 (95.8%)	0.0003
Light and never	5/8 (62.5%)	2/48 (4.2%)	0.0003
Histology			
Adenocarcinoma	4/8 (50.0%)	25/48 (52.1%)	0.68
Squamous cell carcinoma	2/8 (25.0%)	20/48 (41.7%)	0.68
Other	1/8 (12.5%)	3/48 (6.3%)	0.68
TMB status			
High (over 10 Mut/Mb)	5/8 (62.5%)	11/35 (31.4%)	0.67
Low (under 10 Mut/Mb)	3/8 (37.5%)	24/35 (68.6%)	0.67
Gender			
Male	5/8 (62.5%)	36/48 (75.0%)	0.67
Female	3/8 (37.5%)	12/48 (25.0%)	0.67

Abbreviations: *n*, number; PD-L1, programmed death-ligand 1; TMB, tumor mutational burden; Mut/Mb, mutations per megabase. Notes: ^a^ PD-L1 score < 1% is considered negative. ^b^ PD-L1 score > 1% is considered positive.

**Table 6:** Comparing the current study results with findings from other trials.

Study	pCR Rate (%)	MPR Rate (%)	Grade ≥ 3 AE (%)	Treatment Discontinuation (%)	Reference
Current study	36	61	12 (no death)	5.17	
CheckMate 816	24	36.9	33.5 (no death)	10.2	[13]
AEGEAN	17.2	33.3	42.4 (5.7% death)	12	[33]
KEYNOTE-671	18.1	30.2	12.6 (1% death)	12.6	[24]
Neotorch	24.8	48.5	63.4 (0.5% death)	9.4	[32]

Abbreviations: pCR, pathologic complete response; MPR, major pathologic response; AE, adverse events.

## Data Availability

All data generated or analyzed during this study are included in this article. Further inquiries can be directed to the corresponding author.
